# Relationship Between Carotid Intima–Media Thickness, Serum Endocan and Hyaluronic Acid Levels in Multiple Sclerosis

**DOI:** 10.3390/life15091388

**Published:** 2025-09-01

**Authors:** Selcen Duran, Asuman Celikbilek, Ahmet Said Cil, Bilal Ilanbey, Aydan Koysuren, Burc Esra Sahin

**Affiliations:** 1 Department of Neurology, Faculty of Medicine, Kirsehir Ahi Evran University, Kirsehir 40100, Turkey; aydan.koysuren@ahievran.edu.tr (A.K.); besras11@yahoo.com (B.E.S.); 2 Department of Neurology, Sincan Training and Research Hospital, Ankara 06949, Turkey; asunebioglu@yahoo.com; 3 Department of Radiology, Faculty of Medicine, Kirsehir Ahi Evran University, Kirsehir 40100, Turkey; asaidcil@hotmail.com; 4 Department of Medical Biochemistry, Faculty of Medicine, Kirsehir Ahi Evran University, Kirsehir 40100, Turkey; bilalilanbey@hotmail.com

**Keywords:** endothelial dysfunction, carotid intima–media thickness, multiple sclerosis, endocan, hyaluronic acid, vascular pathology

## Abstract

**Background:** Multiple sclerosis (MS) is an immune-mediated neuroinflammatory disorder with a multifactorial etiology involving genetic susceptibility, environmental triggers, and vascular contributions. Carotid intima–media thickness (CIMT) is a significant marker of endothelial dysfunction. Endothelial cell-specific molecule-1 (endocan) and hyaluronic acid, key components implicated in endothelial and vascular remodeling, may significantly contribute to the inflammatory and vascular pathologies observed in MS. We aimed to investigate the relationship between CIMT and endothelial biomarkers, such as endocan and hyaluronic acid, in patients with MS. **Methods:** In this cross-sectional study, 100 patients with relapsing–remitting MS and 56 healthy controls were included. Demographic, clinical, laboratory, and imaging data were documented. CIMT was measured bilaterally using high-resolution B-mode ultrasonography. Serum endocan and hyaluronic acid levels were quantified via enzyme-linked immunosorbent assays. **Results:** MS patients exhibited significantly higher CIMT and serum endocan levels compared with controls (*p* < 0.001). CIMT values were significantly elevated in MS patients, with longer disease duration, higher expanded disability status scale scores, and an older diagnosis age (*p* < 0.05). However, serum endocan and hyaluronic acid levels did not significantly differ between MS subgroups based on disease duration, disability severity, and diagnosis age. Additionally, there was no correlation between CIMT and serum endocan and hyaluronic acid levels in MS patients (*p* > 0.05). **Conclusions:** Increased CIMT and serum endocan levels in MS patients may indicate endothelial dysfunction suggesting vascular involvement in MS. The lack of a correlation between CIMT and endocan and hyaluronic acid levels reveals the complexity of vascular and immune interactions in MS, which needs further research.

## 1. Introduction

Multiple sclerosis (MS) is traditionally considered to be an autoimmune disease; however, accumulating evidence also highlights its immune-mediated and vascular components, including endothelial dysfunction and blood–brain barrier disruption [1,2]. The exact cause of MS and the mechanisms driving its progression remain unclear, although it is widely believed that complex interactions between genetic predisposition and environmental factors play a pivotal role [3]. Furthermore, growing evidence underscores the critical contribution of vascular dysfunction to MS pathophysiology, suggesting a close interplay between the immune and vascular systems in disease progression [4,5]. Recent studies indicate that vascular pathology may be a significant factor contributing to neuronal dysfunction and degeneration in MS [5,6].

Carotid intima–media thickness (CIMT) is widely recognized as a critical indicator for detecting early-stage atherosclerosis [7,8]. CIMT has been extensively studied as a significant marker of vascular health, providing a reliable and non-invasive assessment method through high-resolution B-mode ultrasound, which enables the precise evaluation of arterial wall characteristics [7]. Recent studies have demonstrated increased CIMT in MS, correlating with chronic inflammatory exposure associated with immune pathology [7,8,9,10]. Growing evidence suggests that endothelial dysfunction, chronic inflammation, and impaired cerebral perfusion may contribute to MS progression [2]. Although MS is not primarily a vascular disease, systemic vascular abnormalities, reflected by elevated CIMT, may interact with neuroinflammatory mechanisms by compromising blood–brain barrier (BBB) integrity or contributing to hypoxia [5].

Endothelial cell-specific molecule-1 (endocan) is a soluble dermatan sulfate proteoglycan that modulates the interaction between leukocytes and endothelial cells when the endothelium is activated [11,12,13]. Endocan plays a pivotal role in endothelial dysfunction by functioning as an adhesion molecule, particularly in pathological processes associated with inflammation [14]. Endocan expression is not limited to endothelial cells; glandular tissues, epithelial cells of the kidney and lungs, and cardiac muscle cells also express endocan [15]. It promotes the proliferation and migration of vascular smooth muscle cells, potentially contributing to neointima formation in the progression of atherosclerosis. Recent research indicates its role in neuroinflammation and vascular pathology, positioning it as a potential biomarker of interest in the vascular alterations associated with MS [16,17]. Hyaluronic acid, another endothelium-associated molecule, is a glycosaminoglycan and a major component of the extracellular matrix in soft connective tissues [18]. The breakdown of the glycocalyx at the BBB (of which hyaluronic acid is a key component) has been observed in inflammatory diseases such as sepsis and strokes. This degradation may serve as an early indicator of immune activation, BBB disruption, and disease severity [19,20,21]. Hyaluronic acid plays a dual role in atherosclerosis, promoting vascular smooth muscle cell migration and proliferation while paradoxically exerting protective effects against intimal hyperplasia at high concentrations [22]. The CD-44 receptor to which hyaluronic acid binds plays a dominant role in the ability of immune cells to enter the CNS [23]. Although the potential association between hyaluronic acid and MS remains a hypothesis requiring further investigation, it presents an exciting opportunity to deepen our understanding of endothelial dysfunction in the disease. This line of research may also pave the way for identifying novel, non-invasive biomarkers to monitor disease activity and progression.

Research examining the relationship between CIMT and endothelial biomarkers, particularly endocan and hyaluronic acid, is limited. To date, only three studies have explored this association in autoimmune diseases, including systemic lupus erythematosus, rheumatoid arthritis, and Behçet’s disease [24,25,26]. In MS, which is considered to be a distinct immune-mediated disorder, CIMT [7,8,9,10] and endocan [16,17] have been separately evaluated; however, their combined relationship has not yet been assessed. We hypothesize that increased CIMT in relapsing–remitting MS (RRMS) is associated with elevated serum levels of endocan and hyaluronic acid, reflecting subclinical endothelial dysfunction. Therefore, the aim of this study was to investigate the potential associations between CIMT and these endothelial biomarkers in patients with MS.

## 2. Methods

A total of 100 RRMS patients recruited from an MS outpatient clinic between April 2024 and December 2024 and ranging from 18 to 50 years of age were enrolled in this cross-sectional study. RRMS was diagnosed according to the 2017 McDonald criteria [27]. RRMS patients who were experiencing a relapse at the time of enrolment or within one month of enrolment, as well as those who were pregnant, breastfeeding, or presenting with primary or secondary progressive forms of MS or clinically or radiologically isolated syndromes, were excluded. Additionally, patients with comorbidities such as migraine, rheumatologic, oncologic, endocrinologic, or cardiovascular conditions, including hypertension or elevated blood pressure at screening, and diabetes mellitus that could alter CIMT and endocan and hyaluronic acid levels were excluded.

The control group consisted of 56 hospital staff participants who were similar in age and gender to the patient group. The control group comprised individuals aged 18–55 who did not have neurological diseases such as multiple sclerosis, stroke, or migraine, nor hematological, oncological, rheumatological, or endocrinological diseases, particularly hypertension and diabetes. Individuals with missing data for the patient and control groups were not included in the study.

This study was conducted at the Neurology Clinic of Kirsehir Training and Research Hospital between April 2024 and December 2024, and written informed consent was obtained from all participants in accordance with the Declaration of Helsinki. Ethical approval was obtained from Ahi Evran University Ethics Committee on 26 September 2023 under approval number 2023-16/115. This study was conducted in accordance with the STROBE (Strengthening the Reporting of Observational Studies in Epidemiology) guidelines.

Demographic data (age, sex, and body mass index), disease characteristics (disease duration, diagnosis age, expanded disability status scale [EDSS], plaque burden, relapse count, and time since last relapse), and medication use were documented. Plaque burden was defined as the number of involved regions according to cortical/juxtacortical, periventricular, infratentorial, and spinal involvement using a 1.5 Tesla magnetic resonance imaging (MRI) scan, GE Signa Excite HD 1.5T (GE Healthcare, Milwaukee, WI, USA). Routine laboratory findings included the measurement of serum low-density lipoprotein cholesterol (LDL-C), high-density lipoprotein cholesterol (HDL-C), total cholesterol (TC), triglyceride (TG), vitamin B12, and 25-hydroxyvitamin D levels following an 8 h fasting period.

### 2.1. Carotid Intima–Media Thickness (CIMT)

**Figure 1:**
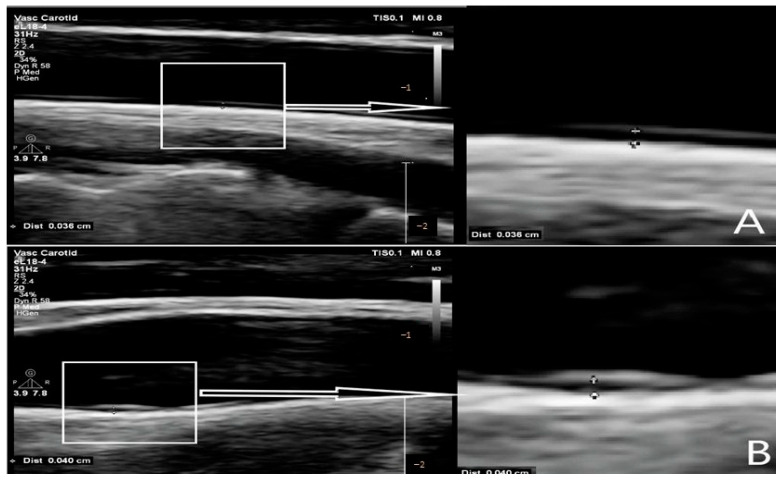
Intimal thickness measurements at the middle portion of the carotid artery (**A**) and at the level of the carotid bulb (**B**).

CIMT was measured bilaterally in 156 participants. All measurements were performed by an experienced neuroradiologist who was manually blinded. Duplex ultrasound (B-mode) was performed using a Philips Affiniti 70 G device equipped with an 18–4 MHz linear probe (Amsterdam, The Netherlands). Participants were placed in a supine position with a slight neck hyperextension to optimize the carotid artery assessment. CIMT was measured at two points, one 10 mm proximal to the common carotid artery (CCA) bifurcation (middle segment of the CCA) and the other at the bulb level, and adhered to recommended guidelines, with measurements taken from the wall of the vessel distal to the probe (Figure 1). Measurements were performed in the diastolic phase.

### 2.2. Enzyme-Linked Immunosorbent Assay (ELISA)

Serum samples for endocan and hyaluronic acid were simultaneously collected (in the morning after an 8 h fast) and stored at −80 °C for later analysis. Serum endocan concentrations were measured using commercially available ELISA kits (Elabscience, Beijing, China) and the sandwich ELISA method. Serum hyaluronic acid concentrations were measured using commercially available ELISA kits (Coon Koon Biotech, Beijing, China) and the sandwich ELISA method. The tests were conducted according to the kit instructions. Optical density was spectrophotometrically measured at 450 nm using a microplate reader (SPECTROstar Nano, BMG Labtech, Ortenberg, Germany).

### 2.3. Statistical Analysis

The categorical and continuous variables are given as numbers (frequency and percentages) and medians and interquartile ranges, respectively. The Kolmogorov–Smirnov test was used to test the normality, and variance homogeneity was assessed using Levene’s test. The Kruskal–Wallis test or the Mann–Whitney U test were performed to compare quantitative data between groups. Chi-squared analyses were used to determine the associations between categorical variables. Analyses were conducted using Statistical Package for the Social Sciences ver. 25.0 (IBM, Armonk, NY, USA), considering *p* < 0.05 to be statistically significant. Multivariable linear regression was used to assess the independent association of serum biomarkers and other covariates with CIMT measured in both right and left common carotid arteries. Independent variables included age, sex, LDL, HDL, total cholesterol, triglycerides, vitamin B12, 25-hydroxyvitamin D, body mass index, and serum endocan and hyaluronan levels.

An a priori power analysis was performed using GPower 3.1 to determine the required sample size. Based on a similar study by Akil et al. [16], which reported a large effect size (Cohen’s d = 0.83) for differences in serum endocan levels between MS patients and controls, we calculated the minimum sample size required to detect a significant effect. With a 5% significance level (α = 0.05) and 80% statistical power (1 − β = 0.80), the analysis indicated that a minimum of 51 participants per group (total *n* = 102) was necessary. Considering potential dropouts and missing data, we included 100 RRMS patients and 56 healthy controls to ensure a robust statistical analysis.

## 3. Results

Age, gender, and body mass index were similar between MS patients and controls (*p* > 0.05). Laboratory findings showed that serum endocan levels were significantly higher in the MS group than in the control group (*p* < 0.001). However, hyaluronic acid levels were similar between the two groups (*p* > 0.05). CIMTs at both measurement points bilaterally were significantly higher in the MS patients compared with the controls (*p* < 0.001). Demographic and laboratory data and CIMT are summarized in Table 1.

In MS patients, the median disease duration was 6 (2–13) years, the diagnosis age was 31 (24–37.8) years, and the EDSS score was 1.5 (1–2.5). Of the patients with MS, 38 (38%) received dimethyl fumarate, 22 (22%) received teriflunomide, 13 received (13%) fingolimod, 12 received (12%) ocrelizumab, and 15 received (15%) other drugs.

In Table 2, MS patients are divided into two groups—less than 5 years and 5 years or more—according to disease duration. CIMTs of the right bulb (*p* = 0.004), right middle segment of the CCA (*p* = 0.006), left bulb (*p* = 0.002), and left middle segment of the CCA (*p* = 0.001) were higher in patients with a disease duration of more than 5 years than those below 5 years. First-line disease-modifying therapies (DMTs) were more common in the group with a diagnosis duration of less than 5 years, while second-line treatments were more common in those with a diagnosis duration of more than 5 years. However, serum endocan and hyaluronic acid levels were similar between the two groups (*p* > 0.05; Table 2).

In Table 3, MS patients are categorized into 3 groups (EDSS < 2, EDSS 2–3, and EDSS ≥ 3) based on the EDSS scores. In the group with high EDSS scores, age, male gender, number of relapses, and disease duration were higher, and DMTs were distributed differently among the groups. CIMTs of the right bulb (*p* = 0.013), right middle segment of the CCA (*p* = 0.004), left bulb (*p* = 0.013), and left middle segment of the CCA (*p* = 0.004) were higher in patients with an EDSS score ≥ 3 compared with the other groups. However, serum endocan and hyaluronic acid levels did not differ between the groups (*p* > 0.005; Table 3).

In Table 4, MS patients are grouped as below or above 30 years, according to their diagnosis age. CIMTs at both measurement points bilaterally were significantly higher in the MS patients diagnosed above 30 years than those diagnosed below 30 years (*p* < 0.001). Serum hyaluronic acid levels were lower in patients diagnosed above 30 years (*p* = 0.028), while serum endocan levels were similar between the groups (*p* > 0.005; Table 4). However, it should be noted that all of these subgroup analyses showed differences in terms of age, BMI, lipid levels, and exposure to treatment, and these differences may have independently affected CIMT.

In the correlation analysis, CIMT exhibited a significant positive correlation with age, body mass index, disease duration, diagnosis age, relapse count, time since the last relapse, EDSS score, LDL-C, TG, and vitamin B12 levels (*p* < 0.05). There was a positive correlation between endocan and hyaluronic acid (r = 0.224; *p* = 0.025). Hyaluronic acid negatively correlated with the time since the last relapse (r = −0.220; *p* = 0.028) and TG (r = −0.275; *p* = 0.006), with a weak negative significance. However, no correlation was found between CIMT and serum endocan and hyaluronic acid levels in MS patients (*p* > 0.05). In the multivariate linear regression analyses including clinical and laboratory parameters (age, sex, body mass index, lipid profile, vitamin levels, serum endocan, and hyaluronic acid), only insignificant changes were noted in the outcomes of the model. The overall pattern of associations was consistent with the findings from the univariate analyses, and no new independent predictors for CIMT were identified.

## 4. Discussion

This study investigated the relationship between CIMT and serum endocan and hyaluronic acid levels in patients with RRMS. The findings indicated that both CIMT and serum endocan levels were significantly increased in MS patients compared with healthy controls, whereas hyaluronic acid levels showed no significant difference. Additionally, no significant correlation was observed between CIMT and endocan. These findings suggest that subclinical vascular changes may be present in MS, although their relationship with systemic endothelial markers remains complex.

Accumulating evidence indicates that dysfunction of the neurovascular unit, a critical regulator of BBB integrity and cerebral blood flow, significantly contributes to the vascular pathology observed in MS. Damage to components of the neurovascular unit, including endothelial cells and pericytes, leads to BBB disruption, neuroinflammation, and the progression of demyelination in MS [6,28]. To further investigate the relationship between neuroinflammation and the vascular hypothesis in MS, this study aimed to assess CIMT alongside the endothelial biomarkers endocan and hyaluronic acid in order to better understand any potential link.

CIMT is known as a significant indicator of vascular health; increased wall thickness may occur in response to chronic inflammatory conditions such as MS. Yuksel et al. reported a significant increase in right CIMT in patients with RRMS compared with healthy controls [7]. Farzan et al. reported that approximately 22% of MS patients exhibited abnormal CIMT measurements and identified a significant association between elevated CIMT and prolonged disease duration, suggesting that vascular remodeling may progress with disease chronicity in MS [10]. Similarly, Najmi et al. demonstrated significantly increased CIMT in MS patients, even after adjusting for age, body mass index, and EDSS scores, thereby supporting the hypothesis that chronic systemic inflammation in MS contributes to endothelial dysfunction, a critical precursor to atherosclerosis [8]. In contrast, Marrie et al. conducted a single study that found no significant difference in average CIMT between MS patients and controls [29]. This was related to the measurement method, as mentioned by Saleh et al. [30]. Consistent with previous studies, our results indicate that CIMT was significantly higher in MS patients compared with the control group across all measurement sites, despite comparable lipid profiles between the groups. Furthermore, CIMT values were elevated in all regions among patients with a longer disease duration, higher EDSS score, and older age at diagnosis. These findings may reflect the potential impact of sustained chronic inflammatory exposure on vascular integrity. Nevertheless, the possible influence of confounding factors such as advanced age and elevated LDL levels in these patients cannot be excluded.

Endocan is implicated in several mechanisms, including vascular permeability, inflammation, and angiogenesis, which may contribute to the pathophysiology of MS. It regulates vascular permeability by upregulating vascular endothelial growth factor-A expression and promoting endothelial activation, thereby facilitating immune cell infiltration into the CNS [12,13]. Although endocan has not yet been established as a clinical biomarker, research on its potential utility in detecting subclinical vascular dysfunction in MS remains limited. Atalar et al. reported significantly elevated serum endocan levels in an MS group with a comorbidity rate of 45.3% compared with controls; however, serum endocan levels did not significantly differ between MS subgroups [17]. Similarly, Akil et al. found that serum endocan levels were significantly higher in MS patients than in controls, with notably elevated levels during relapse periods compared with remission, suggesting its potential as a marker for disease activity and relapse dynamics in MS [16]. Consistent with these findings, our results demonstrated significantly increased serum endocan levels in the MS group relative to the controls, which may reflect enhanced vascular permeability in MS patients.

There are limited studies examining the relationship between CIMT and endocan, with existing studies predominantly focusing on autoimmune diseases. One study reported elevated serum endocan levels in patients with Behçet’s disease compared with controls, demonstrating a positive correlation between endocan levels, CIMT, and disease activity [26]. Another study identified a significant positive correlation between serum endocan and CIMT in individuals with systemic lupus erythematosus [25]. A third investigation involving patients with rheumatoid arthritis found higher serum endocan levels and increased CIMT in the patient group compared with the controls [24]. Consistent with these findings, our study observed elevated CIMT and serum endocan levels in MS compared with the controls; however, no significant correlation between these variables was detected.

The lack of a correlation between serum endocan or hyaluronic acid levels and CIMT may reflect fundamental differences in the anatomical and physiological domains represented by these markers. Multiple mechanisms may contribute to the lack of correlation between serum endocan levels and CIMT in MS. MS is increasingly recognized not as a classical autoimmune disease, but rather as an immune-mediated disorder with multifactorial origins, including genetic susceptibility, environmental triggers, and neurovascular dysfunction. Unlike systemic autoimmune diseases, where widespread inflammation leads to generalized endothelial activation, the immune response in MS is largely compartmentalized within the central nervous system. This includes localized endothelial dysfunction, BBB disruption, and perivascular inflammation—features that may not be mirrored in systemic biomarker profiles. This localized inflammation may not necessarily result in systemic vascular changes that are detectable by CIMT. In the context of MS, which is increasingly recognized as a compartmentalized CNS disease, peripheral serum biomarkers may not accurately represent intrathecal vascular or immune processes. DMTs have demonstrated efficacy in stabilizing the BBB in individuals with MS by reducing immune cell trafficking and preserving endothelial integrity. This therapeutic intervention may reduce central nervous system inflammation [31]. Furthermore, the modulation of endothelial activation could lead to a decrease in systemic endothelial biomarker signals, such as endocan, which may subsequently diminish any observable correlation with CIMT in patients undergoing treatment. Although CIMT is likely indicative of chronic structural vascular alterations, endocan may serve as a dynamic marker of acute endothelial activation. This assertion is supported by our findings, which demonstrate that, unlike CIMT, serum endocan levels did not exhibit significant differences across subgroups categorized by disease duration, EDSS score, or age at diagnosis. The relatively stable levels of endocan across various MS subtypes may also reflect the inclusion of patients in the relapse-free phase, during which systemic inflammatory activity is likely to be low.

Hyaluronic acid is a representative glycosaminoglycan present in the glycocalyx that can indicate endothelial glycocalyx degradation [32,33]. This layer serves as the first defensive layer of the BBB and its disruption causes endothelial permeability, allowing inflammatory cytokines, immune cells, and antibodies to leak into the CSF, thereby worsening inflammation and disease severity [19,20,33]. Hyaluronic acid may play a key role in MS pathogenesis because its deposition increases in response to injury or inflammation, particularly in demyelinated lesions, where it promotes T-cell, microglial, and macrophage activation [32,34]. Hyaluronic acid fragments, generated by elevated hyaluronidase activity [21], sustain astrocyte reactivity and local immune responses, whereas they inhibit oligodendrocyte maturation, thus preventing myelin repair and facilitating inflammatory cell infiltration into the CNS in MS patients [32,34]. A single study by Zhang et al. found higher serum and CSF hyaluronic acid levels in an MS and neuromyelitis optica group than in the control group, and this correlated with disease severity [33]. Contrarily, we found similar serum hyaluronic acid levels between the two groups. This may be explained by the patient selection criteria because we included a heterogeneous RRMS patient group with different disease durations, EDSS scores, and diagnosis ages, while Zhang et al. included MS patients with high EDSS scores [33]. Regarding subgroups, serum hyaluronic acid levels were significantly higher in patients diagnosed below 30 years of age than in those diagnosed above 30. This may due to the excessive inflammation in the early phase of the disease compared with the later stages. Contrary to previous findings, our study did not reveal a significant difference in serum hyaluronic acid levels between MS patients and the control group. Our results suggest that systemic measurements may not fully reflect the local neuroinflammatory roles of hyaluronic acid within the central nervous system. To better elucidate the pathophysiological role of hyaluronic acid, future research involving CSF analyses, animal models, or in vitro studies may be necessary.

This study has several limitations. First, its single-center design and relatively small sample size may have limited the generalizability of the findings. However, the inclusion of an a priori power analysis supports the adequacy of the sample size to detect meaningful associations. Second, the cross-sectional design precluded any determination of causality. Third, the absence of inflammatory serum markers, such as interleukin-6, could limit a more comprehensive understanding of MS pathophysiology. Fourth, measuring hyaluronic acid levels only in serum, rather than in CSF, may have influenced the results. Fifth, including only relapse-free patients with RRMS may have restricted the ability to detect acute changes in endothelial activation, which could be more pronounced during disease exacerbation. Sixth, the uneven distribution of disease-modifying therapies among patient subgroups may have affected both CIMT and endothelial biomarker levels. Lastly, CIMT subgroup comparisons based on disease duration and EDSS scores may have been unreliable due to significant differences in known vascular risk factors such as age, BMI, lipid profiles, and treatment regimens.

## 5. Conclusions

To the best of our knowledge, this is the first study to investigate the relationship between CIMT and endocan and hyaluronic acid levels in MS patients. Compared with the controls, we found higher CIMT and serum endocan levels in the RRMS group, which may support evidence of endothelial dysfunction and suggests vascular involvement in MS. However, we did not observe a correlation between CIMT and endocan and hyaluronic acid levels, highlighting the complexity of vascular and immune interactions in MS, indicating the need for further research.

## Figures and Tables

**Table 1:** Demographic data, laboratory findings, and carotid intima–media thickness in multiple sclerosis patients and the control group (*n* = 156).

	Patient (*n* = 100)	Control (*n* = 56)	*p*-Value
DEMOGRAPHICS			
Age (years)	37.5 (32–47)	36.5 (29.3–42)	0.158
Female gender	76 (76%)	43 (76.8%)	0.912
Body mass index (kg/m^2^)	24.5 (22.3–27.3)	25.1 (22.7–26.6)	0.904
Smoking status	20 (58.8%)	14 (41.2%)	0.468
LABORATORY FINDINGS			
LDL-C (mg/dl)	96.5 (76.3–122.8)	93 (78.3–118)	0.536
HDL-C (mg/dl)	52.5 (47–64.5)	46 (40–57)	0.009
TC (mg/dl)	169 (149.3–205.8)	164 (145–185.5)	0.247
TG (mg/dl)	97.5 (64.3–140)	91.5 (69.8–143.8)	0.907
Vitamin B12 (pg/mL)	343.5 (277–429.5)	350.5 (278.3–427.5)	0.836
25-hydroxyvitamin D (pg/mL)	17 (10–27)	17 (10.5–22)	0.531
Endocan (pg/mL)	36.9 (24.6–51.7)	23 (14.7–33.8)	<0.001
Hyaluronic acid (pg/mL)	430.5 (268–643.8)	404 (152–691.8)	0.305
CAROTID INTIMA–MEDIA THICKNESS			
❖ Right side			
The bulb	0.43 (0.36–0.59)	0.33 (0.29–0.39)	<0.001
The middle segment of the CCA	0.37 (0.32–0.46)	0.31 (0.26–0.36)	
❖ Left side			
The bulb	0.43 (0.36–0.60)	0.35 (0.29–0.39)	<0.001
The middle segment of the CCA	0.37 (0.33–0.47)	0.32 (0.27–0.37)	

LDL-C: low-density lipoprotein cholesterol; HDL-C: high-density lipoprotein cholesterol; TC: total cholesterol; TG: triglyceride; CCA: common carotid artery.

**Table 2:** Comparison of data according to the disease duration of patients with multiple sclerosis (*n* = 100).

	1–4 Years (*n* = 48)	≥5 Years (*n* = 52)	*p*-Value
DEMOGRAPHICS			
Age (years)	34 (27.3–42.5)	43 (35.3–52)	<0.001
Female gender	36 (75%)	40 (76.9%)	0.822
Body mass index (kg/m^2^)	23.5 (22.0–27.4)	24.9 (22.8–27.0)	0.201
Smoking status	10 (50%)	10 (50%)	0.841
DISEASE CHARACTERISTICS			
Diagnosis age (years)	31 (26–38.8)	29.5 (23.3–36.8)	0.240
Plaque burden	3 (2–3)	3 (2–3)	0.918
Relapse count	1 (1–2)	3 (2–6)	<0.001
Time since the last relapse (month)	15 (6–36)	36 (12–72)	
EDSS	1.5 (1–2)	2 (1.125–3)	
MEDICATION			
Dimetil fumarat	30 (62.5%)	8 (15.4%)	<0.001
Teriflunamid	11 (22.9%)	11 (21.2%)	
Fingolimod	1 (2.1%)	12 (23.1%)	
Ocrelizumab	3 (6.3%)	9 (17.3%)	
Other	3 (6.3%)	9 (17.3%)	
CAROTID INTIMA–MEDIA THICKNESS			
❖ Right side			
The bulb	0.41 (0.34–0.50)	0.47 (0.38–0.64)	0.004
The middle segment of the CCA	0.34 (0.32–0.43)	0.41 (0.34–0.48)	0.006
❖ Left side			
The bulb	0.39 (0.34–0.49)	0.47 (0.39–0.64)	0.002
The middle segment of the CCA	0.34 (0.32–0.40)	0.41 (0.35–0.49)	0.001
LABORATORY FINDINGS			
Endocan (pg/mL)	40.3 (25.9–52.6)	31.0 (21.2–50.7)	0.108
Hyaluronic acid (pg/mL)	461.5 (277.8–691.8)	407.5 (252.5–562.3)	0.456

EDSS: expanded disability status scale; CCA: common carotid artery.

**Table 3:** Comparison of data according to the expanded disability status scale (EDSS) scores of patients with multiple sclerosis (*n* = 100).

	EDSS < 2 (*n* = 53)	EDSS 2–2.5 (*n* = 29)	EDSS ≥ 3 (*n* = 18)	*p*-Value
DEMOGRAPHICS				
Age (years)	34 (27–40)	44 (34.5–51)	47.5 (36.75–54)	<0.001
Female gender	41 (84.9%)	21 (72.4%)	10 (55.6%)	0.036
Body mass index (kg/m^2^)	24.1 (21.9–27.5)	24.7 (22.8–28.5)	25.8 (23.0–26.7)	0.512
Smoking status	12 (60%)	5 (25%)	3 (15%)	0.781
DISEASE CHARACTERISTICS				
Diagnosis age (years)	29 (22–34)	33 (25.5–41)	32.5 (23.8–38.5)	0.098
Plaque burden	3 (2–3)	3 (2–3)	3 (2–3.25)	0.218
Relapse count	2 (1–3)	2 (2–3)	5 (1.75–10)	0.001
Time since the last relapse (month)	24 (6–42)	24 (12–54)	36 (12–60)	0.410
Disease duration (years)	3 (1–7)	7 (4–14.5)	15 (6.5–17)	<0.001
MEDICATION				
Dimetil fumarat	30 (56.6%)	4 (13.8%)	4 (13.8%)	<0.001
Teriflunamid	10 (18.9%)	11 (37.9%)	1 (5.6%)	
Fingolimod	5 (9.4%)	4 (13.8%)	4 (22.2%)	
Ocrelizumab	2 (3.8%)	3 (1.3%)	7 (38.9%)	
Other				
CAROTID INTIMA–MEDIA THICKNESS				
❖ Right side				
The bulb	0.38 (0.34–0.47)	0.48 (0.38–0.63)	0.52 (0.42–0.63)	0.013
The middle segment of the CCA	0.34 (0.32–0.4)	0.41 (0.34–0.48)	0.44 (0.36–0.51)	0.004
❖ Left side				
The bulb	0.38 (0.34–0.66)	0.47 (0.38–0.62)	0.54 (0.42–0.63)	0.013
The middle segment of the CCA	0.34 (0.32–0.4)	0.42 (0.34–0.47)	0.43 (0.36–0.52)	0.004
LABORATORY FINDINGS				
Endocan (pg/mL)	40.5 (25.0–52.2)	35.9 (22.1–44.8)	33.9 (25.6–60.7)	0.667
Hyaluronic acid (pg/mL)	458 (283–684.5)	393 (217–555)	425.5 (272–775.5)	0.324

EDSS: expanded disability status scale; CCA: common carotid artery.

**Table 4:** Comparison of data according to a diagnosis age below or above 30 years for patients with multiple sclerosis (*n* = 100).

	<30 Years (*n* = 49)	≥30 Years (*n* = 51)	*p*-Value
DEMOGRAPHICS			
Age (years)	32 (26.5–37.5)	46 (37–52)	<0.001
Female gender	39 (79.6%)	37 (72.5%)	0.410
Body mass index (kg/m^2^)	23.4 (21.7–25.5)	25.8 (23.1–28.4)	0.001
Smoking status	9 (45%)	11 (55%)	0.689
DISEASE CHARACTERISTICS			
Plaque burden	3 (2–3)	3 (2–3)	0.929
Relapse count	2 (1.5–4.5)	2 (1–3)	0.199
Time since the last relapse (month)	24 (6–54)	24 (12–36)	0.950
Disease duration (years)	7 (2.5–14.5)	4 (1–3)	0.145
EDSS	1.5 (1–2.25)	2 (1–2.5)	0.564
CAROTID INTIMA–MEDIA THICKNESS			
❖ Right side			
The bulb	0.38 (0.34–0.44)	0.55 (0.42–0.65)	<0.001
The middle segment of the CCA	0.33 (0.32-0.36)	0.44 (0.36–0.51)	
❖ Left side			
The bulb	0.37 (0.34–0.44)	0.55 (0.43–0.65)	<0.001
The middle segment of the CCA	0.33 (0.32–0.37)	0.44 (0.36–0.51)	
LABORATORY FINDINGS			
Endocan (pg/mL)	40.52 (25.39–52.18)	33.86 (24.37–51.45)	0.291
Hyaluronic acid (pg/mL)	495 (327–799)	392 (231–562)	0.028

EDSS: expanded disability status scale; CCA: common carotid artery.

## Data Availability

The dataset is available upon request from the authors.

## Abbreviations

MS	Multiple Sclerosis
BBB	Blood–Brain Barrier
CIMT	Carotid Intima–Media Thickness
Endocan	Endothelial Cell-Specific Molecule-1
CNS	Central Nervous System
RRMS	Relapsing–Remitting Multiple Sclerosis
EDSS	Expanded Disability Status Scale
MRI	Magnetic Resonance Imaging
LDL-C	Low-Density Lipoprotein Cholesterol
HDL-C	High-Density Lipoprotein Cholesterol
TC	Total Cholesterol
TG	Triglycerides
CCA	Common Carotid Artery
ELISA	Enzyme-Linked Immunosorbent Assay
CSF	Cerebro Spinal Fluid
